# A novel rapid formulation of intravenous dantrolene: Preclinical assessment

**DOI:** 10.1097/EA9.0000000000000059

**Published:** 2024-09-10

**Authors:** Richard H. Ng Kwet Shing, Samuel L. Smith

**Affiliations:** From Norgine, Harefield, UK

Editor,

Malignant hyperthermia, a life-threatening anaesthetic emergency, requires urgent intervention.^1^ The indicated treatment, dantrolene sodium, has poor water solubility.^1^ The formulation available in Europe, DANTRIUM^®^ IV/DANTROLEN™ IV (Norgine, Harefield, UK), hereinafter referred to as DANTRIUM, comes in 20 mg vials (with mannitol and sodium hydroxide) requiring 60 ml water, shaking and filtering, which is time-consuming.^2^

A new formulation, NPJ5008, supplied in vials of 120 mg dantrolene with 2-hydroxypropyl-beta-cyclodextrin (HP-β-CD) and polyethylene glycol (PEG) 3350, to be dissolved in 20 ml water, yields a solution of 16.5-fold greater concentration than DANTRIUM, with reduced time and effort for preparation and administration.^3^

**Fig. 1:**
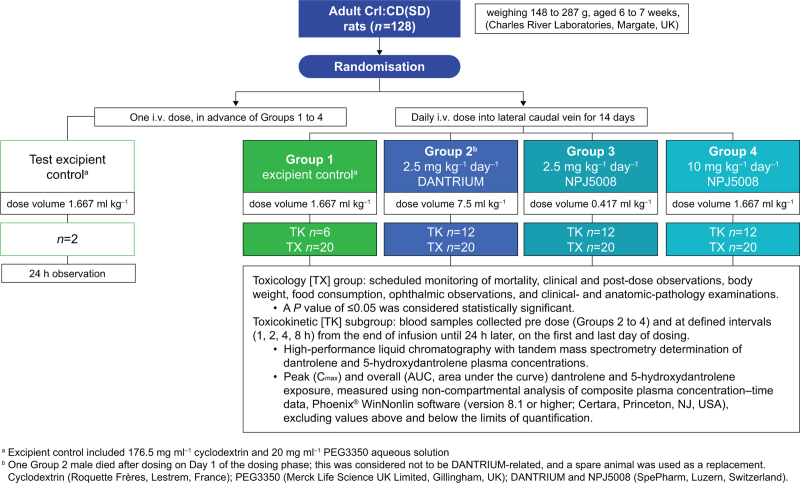
Study design of repeat administration investigation in rats.

To evaluate the suitability of NPJ5008 for clinical testing, standard preclinical studies addressed the product's in-vitro plasma protein-binding and haemolysis potential, and in-vivo repeat-dose toxicity and toxicokinetic profile in rats, compared with DANTRIUM at clinically relevant concentrations. The toxicity/toxicokinetic investigation in rats (Fig. 1) was Good Laboratory Practice-compliant (Home Office Project Licence Number: P044BE9B9) and adhered to the Animals (Scientific Procedures) Act 1986. Ethical approval was provided by the Animal Welfare and Ethical Review Body, Covance, Harrogate, UK, lead Timothy Jameson, on 20 January 2020, and a local ethical review was maintained by Covance (now Labcorp) responsible/designated personnel (protocol number 8418863, approved for use 7 January 2020). Study period was from 7 January 2020 to 18 December 2020. Statistical pairwise comparison between Group 1 controls and Groups 2 to 4 (separately by sex) evaluated toxicity (Levene's test for variance equality, rank transformation if needed, and ANOVA followed by Dunnett's test; SAS, Cary, North Carolina, USA, version 9.4).

Similar mean dantrolene plasma-protein unbound fractions of 5 to 8% showed the formulation change had no effect on protein binding. Mean haemoglobin release values of 4% or less, with negligible free-iron levels, excluded haemolytic potential in either formulation (data not shown).

Preclinical toxicity analysis revealed that no treatment-related mortality, ophthalmic or clinical condition changes occurred.

**Fig. 2:**
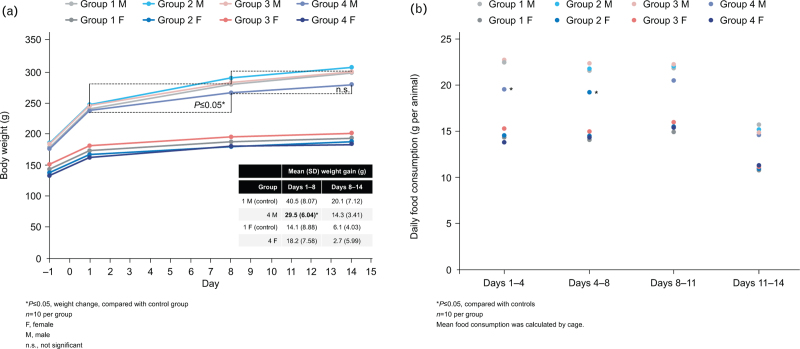
In-vivo toxicity repeat administration study in rats.

Group 4 males gained 27.6% less weight than controls over the dosing period, with significantly less food consumption and weight gain over Days 1 to 8 (*P* ≤ 0.05, Fig. 2).

(a) Weight and statistical comparison of weight change and (b) food consumption during the study. Group 1, excipient control; Group 2, DANTRIUM (2.5 mg kg^−1^ day^−1^); Group 3, NPJ5008 (2.5 mg kg^−1^ day^−1^); Group 4, NPJ5008 (10 mg kg^−1^ day^−1^).

All post–dose observations occurred only over Days 1 to 8, resolved within 2 h and included:

(1) Group 4: ataxia (*n* = 19), low carriage (*n* = 20), mildly splayed gait (*n* = 1) and mildly (*n* = 18) to moderately (*n* = 5) decreased activity
(2) Group 2: gait ataxia (*n* = 2)
(3) Group 3: gait ataxia (*n* = 1) and low carriage (*n* = 1)

Organ weight observations fell within normal biological ranges with small but significant (*P* ≤ 0.05) decreases compared with controls in kidney:body weight (7 to 8%, Group 2 and 3 females and Group 2 males) and kidney:brain weight (11%, Group 2 females) only.

Tissues were macroscopically unremarkable. Microscopic findings were generally infrequent and minor: vacuolated renal tubular epithelial cells occurred in Groups 1 and 4, vacuolated alveolar macrophages in males of Groups 1 and 4, and vacuolated Kupffer cells in one Group 3 female.

The following significant (*P* ≤ 0.05) clinical pathology changes occurred compared with controls:

(1) Group 4 males, higher cholesterol and increased urine volume
(2) Group 3 females, higher cholesterol and triglycerides
(3) Group 4 females, lower globulin and phosphate
(4) Groups 2 to 4 females, decreased enzymatic creatinine

Toxicokinetic data revealed sex differences (less than twofold) in dantrolene maximum observed concentration (C_max_) and area under the concentration–time curve from Hour 0 to 24 (AUC_0–24.083_) values on Days 1 and 14, therefore sexes were analysed separately (Table 1). Comparing equivalent doses of NPJ5008 and DANTRIUM: dantrolene C_max_ and AUC_0–24.083_ exposures, although somewhat higher with DANTRIUM, were similar; the metabolite 5-hydroxydantrolene C_max_ and AUC_0–24.083_ values were similar; the metabolite:parent ratios were similar; and the accumulation ratios for dantrolene and 5-hydroxydantrolene after multiple dosing were similar, with higher C_max_ and AUC_0–24.083_ values in males than in females. The relative bioavailability of dantrolene (F_rel_% AUC_0–24.083_ NPJ5008:DANTRIUM) was 65.3 and 89.8% on Day 1, and 85.5 and 68.3% on Day 14, for males and females, respectively. With increasing NPJ5008 dose level, C_max_ and AUC_0–24.083_ values increased, with a greater-than-dose proportional relationship in AUC values for both dantrolene and 5-hydroxydantrolene, and in C_max_ values for dantrolene only.

**Table 1:**
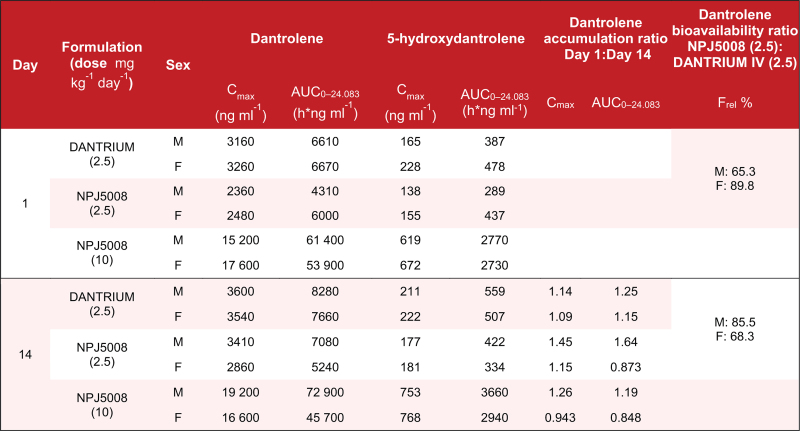
In-vivo toxicokinetic repeat administration study in rats

Data indicate that the two formulations have similar properties and are consistent with historic intravenous dantrolene data (Norgine, data on file). In terms of C_max_ and AUC, dantrolene exposure was higher for DANTRIUM than for an equivalent dose of NPJ5008, but values were generally similar. Similar metabolite:parent ratios indicate that metabolising enzymes can access dantrolene regardless of formulation. This suggests that dantrolene in NPJ5008 should be available to interact with its therapeutic target.

NPJ5008 demonstrated a similar toxicity profile to DANTRIUM. Transient muscle weakness-related observations, primarily in Group 4, were consistent with the desired mechanism of action of dantrolene.^4^ Increased urine volume in Group 4 suggested impaired renal retention. Reduced weight gain in Group 4 males, coinciding with decreased food consumption, may have resulted from the pharmacological action of dantrolene. Anatomic and clinical pathology was indistinguishable between groups. Any significant differences were small, inconsistent between sexes and between formulations, not dose-dependent or unaccompanied by in-life or pathological changes, and attributable to normal biological variation. An increased incidence of low-grade vacuolated cells, primarily in the kidney tubule, among Group 1, 3 and 4 animals was likely caused by the HP-β-CD component, as findings are consistent with published data of a transient, reversible cyclodextrin class effect following prolonged exposure in rodents.^5^ HP-β-CD appears well tolerated in humans, with no reported adverse effects on kidneys or other organs following oral or intravenous administration.^5^ On the basis of the treatment-related findings in this study, the no observed adverse effect level was 2.5 mg kg^−1^ day^−1^ for NPJ5008 and at least 2.5 mg kg^−1^ day^−1^ for DANTRIUM.

A limitation of the standard preclinical toxicology approach adopted here is that specialised investigations such as ototoxicity are precluded. High doses of HP-β-CD have been associated preclinically with auditory hair cell loss.^6^ The relevance of this is unclear, as methods of administration and dosages over time differ from the current study.

These results indicate NPJ5008's suitability for clinical testing, and it is expected to be well tolerated and effective in malignant hyperthermia. The new formulation permits a sixfold higher amount of dantrolene to be timely reconstituted in one-third of solvent volume, which may have clinical relevance.
